# Identifying Pupylation Proteins and Sites by Incorporating Multiple Methods

**DOI:** 10.3389/fendo.2022.849549

**Published:** 2022-04-26

**Authors:** Wang-Ren Qiu, Meng-Yue Guan, Qian-Kun Wang, Li-Liang Lou, Xuan Xiao

**Affiliations:** School of Information Engineering, Jingdezhen Ceramic Institute, Jingdezhen, China

**Keywords:** pupylation, multiple features, post-translational modification, chi-square test, word embedding

## Abstract

Pupylation is an important posttranslational modification in proteins and plays a key role in the cell function of microorganisms; an accurate prediction of pupylation proteins and specified sites is of great significance for the study of basic biological processes and development of related drugs since it would greatly save experimental costs and improve work efficiency. In this work, we first constructed a model for identifying pupylation proteins. To improve the pupylation protein prediction model, the KNN scoring matrix model based on functional domain GO annotation and the Word Embedding model were used to extract the features and Random Under-sampling (RUS) and Synthetic Minority Over-sampling Technique (SMOTE) were applied to balance the dataset. Finally, the balanced data sets were input into Extreme Gradient Boosting (XGBoost). The performance of 10-fold cross-validation shows that accuracy (ACC), Matthew’s correlation coefficient (MCC), and area under the ROC curve (AUC) are 95.23%, 0.8100, and 0.9864, respectively. For the pupylation site prediction model, six feature extraction codes (i.e., TPC, AAI, One-hot, PseAAC, CKSAAP, and Word Embedding) served to extract protein sequence features, and the chi-square test was employed for feature selection. Rigorous 10-fold cross-validations indicated that the accuracies are very high and outperformed its existing counterparts. Finally, for the convenience of researchers, PUP-PS-Fuse has been established at https://bioinfo.jcu.edu.cn/PUP-PS-Fuse and http://121.36.221.79/PUP-PS-Fuse/as a backup.

## 1 Introduction

Pupylation is a kind of prokaryotic ubiquitin-like protein (Pup), a posttranslational protein modification (PTM) that occurs in actinomycetes, and has made a great contribution to the life process of many cells (1, 2). Ubiquitylation is one of the most common PTM modifications (3). In eukaryotes, ubiquitylation modification plays an important role in DNA repair, transcription regulation, control signal transduction, endocytosis, and sorting (4); research has shown that ubiquitylation modification is closely related to human health, such as lung cancer, breast cancer, type II diabetes, and other complex diseases (5–8). Pupylation is similar to ubiquitin in that Pup is attached to specific lysine residues. Since the PTM small protein modification was originally discovered in prokaryotes, the Pup in Mycobacterium tuberculosis (Mtb) plays an important role in the selection of protein degradation (5).

To better understand the biological mechanism of pupylation, the basic goal and fundamental task is to accurately and effectively predict the pupylation proteins and sites. For identifying PTM proteins, to the best of our knowledge, Qiu is the first one to have tried to identify phosphorylated (9) and acetylated (10) proteins, and nobody has done a similar work on pupylation protein until now. For a predictive analysis of pupylation sites, Liu proposed a GPS-PUP predictor for predicting pupylation sites with a group-based prediction system (GPS) method (11). Tung developed an iPUP predictor that implemented the support vector machine (SVM) algorithm with the composition of pairs of k-space amino acids (CKSAAP) (12). Chen designed a predictor called PupPred based on support vector machines (SVM), in which amino acid pairs were used to encode lysine-centered peptides (13). Hasan established a web server named pbPUP (14), which was a profile-based feature method to predict pupylation sites. Recently, FN Auliah developed PUP-Fuse web server for predicting pupylation sites (15); this algorithm was based on a variety of sequence features to predict pupylation sites. Although these algorithms could output higher specificity, their sensitivity scores are much lower.

**Figure 1:**
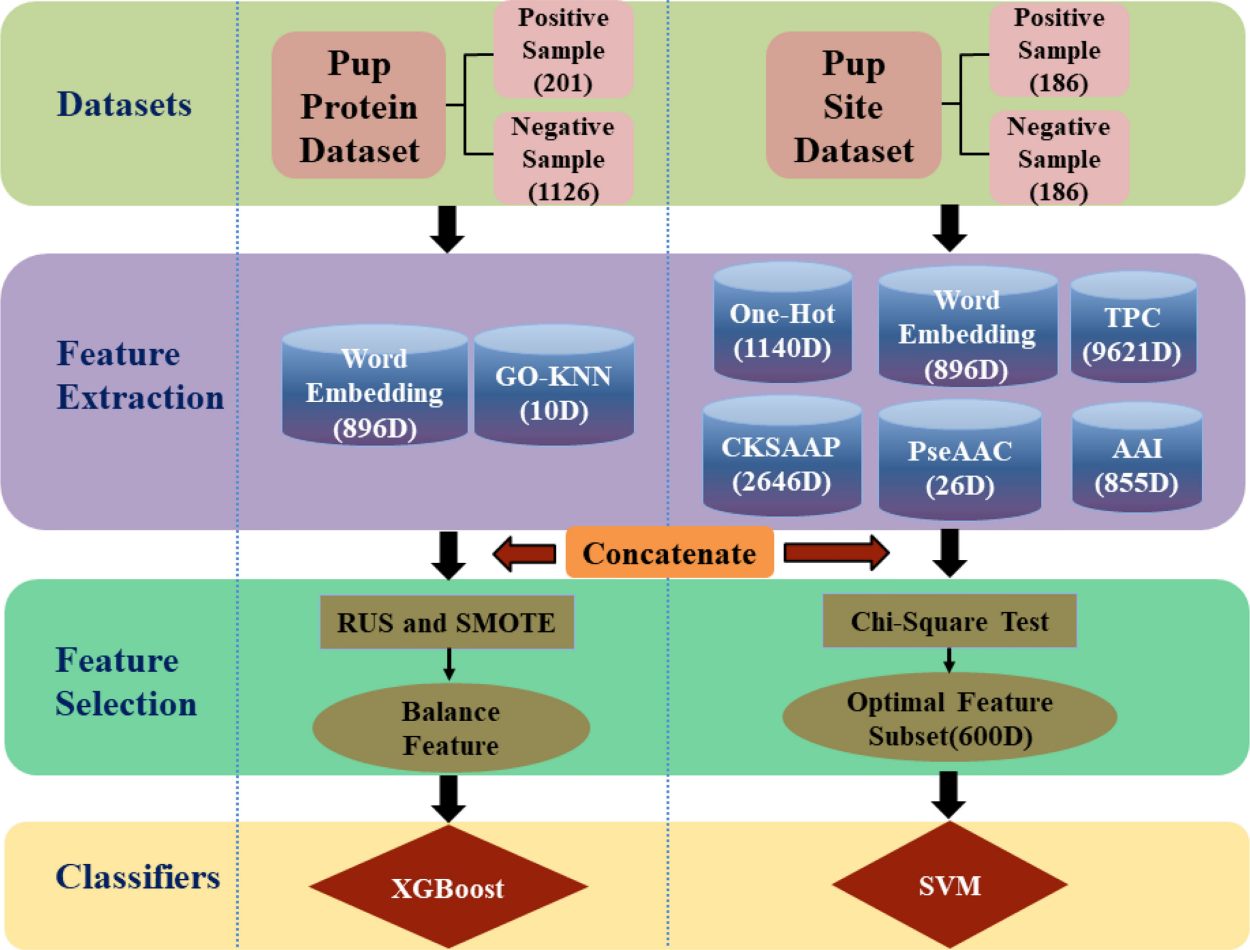
The framework of PUP-PS-Fuse (rounded squares represent data sets, cylinders represent feature extraction methods, rectangles and ellipses represent feature selection methods, and diamonds represent classifiers. RUS is the abbreviation of Random Under-sampling and SMOTE is the abbreviation of Synthetic Minority Over-sampling).

In this work, a framework has been developed for predicting pupylation proteins and sites named as PUP-PS-Fuse, shown in **Figure 1**. In predicting the pupylation protein model, the KNN scoring matrix, the Word Embedding model (16–18), the Synthetic Minority Oversampling Technique(SMOTE) (19), and Random Under-sampling(RUS) (20) were applied to enhance the operation engine. Moreover, in the pupylation site prediction model, TPC (15, 21), AAI (22, 23), One-Hot (24), PseAAC (25, 26), CKSAAP (21, 27, 28), and Word Embedding (16–18) were used for feature extraction, and the chi-square test (15, 29, 30) was used to reduce the dimensionality of the feature space. Both these two models were verified with 10-fold cross-validation and compared with other existing predictors, the performance proved that this work is promising for the issue.

## 2 Materials

### 2.1 Datasets for Predicting Pupylation Proteins

In this work, the negative samples were collected from UniProKB(2021_4), and the positive sample set was composed of 35 pupylation proteins collected from UniProKB and 233 pupylation proteins from PupDB (31). At least one pupylation site must exist in any positive protein sequence, and none of the pupylation sites must appear in the negative samples. A given protein sequence can be expressed as P=*R*_1_ *R*_2_ *R*_3_...*R_i_*...*R*_L_; here, *R_i_* represents the *i*th amino acid residue, and *L* represents the length of the protein sequence.

In order to make the results more rigorous, CD-HIT was used to remove 30% of the redundancy from 268 positive sampling as and 1,463 negative samples. Finally, 201 positive samples and 1,126 negative samples were collected for the proposed benchmark with a positive–negative ratio of 1:5.6.

### 2.2 Datasets for Predicting Pupylation Sites

This article used the same data set as that of Aulia (15). The data set was retrieved and obtained from the publications of PupDB (31) and contained 233 pupylation proteins which were subject to a cutting of redundancy treatment to remove those sequences that had ≥80% pairwise sequence identity with any other. After strictly following the aforementioned procedures, the training set consists of 186 amino acid fragments with pupylation site as positive samples and 372 negative samples without any pupylation site. As a result, the positive–negative ratio is 1:2. Since the imbalance of the data will affect the prediction results of the model, we balanced the training set with a positive–negative ratio of 1:1 (186 positive samples and 186 negative samples) by randomly deleting negative samples. The test set is composed of 87 positive samples and 191 negative samples by randomly extracting from the benchmark data set. **Table 1** summarizes the data sets for predicting pupylation proteins and pupylation sites.

**Table 1:** Data set for prediction of pupylation protein and pupylation site.

Datasets	Positive	Negative	Ratio
Pupylation proteins	201	1126	1:5.6
Pupylation site training	186	186	1:1
Pupylation site test	87	191	1:2.2

Positive represents the number of positive samples, and Negative represents the number of negative samples.

In order to formulate the pupylation site sequence in more detail and more comprehensively, the sequence fragment of the potential pupylation site can be expressed in the form of formula (1):


(1) $$\theta_{\delta }\left(K\right)=R_{1}R_{2}-R_{\delta -1}R_{\delta }KR_{\delta +1}R_{\delta +2}\cdots R_{2\delta -1}R_{2\delta }$$

Where *R*_1_ to *R*_δ_ represent the amino acid residues on the left of *K*, *R*_δ+1_ to *R_2_*_δ_ represent the amino acid residues on the right of *K*, δ is an integer, and the middle *K* means *Lysine* (32). In addition, the peptide sequence θ_δ_(*K*) can be divided into $\theta_{\delta }^{+}\left(K\right)$ and $\theta_{\delta }^{-}\left(K\right)$ (see formula (2)), where $\theta_{\delta }^{+}\left(K\right)$ represents a pupylation protein sequence fragment whose center point is *K*, and $\theta_{\delta }^{-}\left(K\right)$ denotes non-pupylation protein sequence fragments whose center point is *K*. The sliding window method was used to segment pupylation protein sequences with different window sizes. Judging from the analysis of the pupylation protein sequence preferred by FN Aulia et al. (15), it can be seen that the prediction is the best when the window size is 57 with δ = 28.

When the sequence fragments were divided, in order to make the site sequence equal in length, the missing amino acids were filled in with *X* residues. As a result, the pupylation site data set adopts the form of formula (2):


(2) $$\theta_{\delta }\left(K\right)=\theta_{\delta }^{+}\left(K\right)\cup \theta_{\delta }^{-}\left(K\right)$$

Among them, the subset of positive samples $\theta_{\delta }^{+}\left(K\right)$ represents a true pupylation site segment with *K* at its center, and the subset of negative samples $\theta_{\delta }^{-}\left(K\right)$ represents the false pupylation site fragment.

## 3 Feature Extraction and Methods

### 3.1 Feature Extraction Methods for Predicting Pupylation Proteins

The basic step for predicting pupylation protein is to extract features of the protein sequence, and it is a key step that affects the effectiveness of the prediction model. When predicting pupylation protein, we chose GO-KNN (10) and Word Embedding coding schemes to extract protein sequence information.

#### 3.1.1 GO-KNN

GO-KNN (10) is based on the KNN scoring matrix of functional domain GO annotations to extract features. In this study, we need to obtain the GO information of all proteins. For a protein without any GO information, we replace it with GO terms of its homologous protein and then calculate the distance between any two protein sequences. Taking protein *R*_1_ and *R*_2_ as example, their GO annotations can be expressed by $R_{GO}^{1}=\left\{GO_{1}^{1},GO_{2}^{1},\cdots ,GO_{M}^{1}\right\}$ and $R_{GO}^{2}=\left\{GO_{1}^{2},GO_{2}^{2},\cdots ,GO_{N}^{2}\right\}$ , $GO_{i}^{1}$ and $GO_{i}^{2}$ represent the *i*th GO of the proteins *R*_1_ and *R*_2_, respectively, and *M* and *N* are the numbers of their GO terms. The feature extraction steps are listed as follows:

(a). Calculating the distance between two proteins, as in formula (3).


(3) $$\mathrm{Distance}\left(R_{1},R_{2}\right)=1-\frac{\left\lfloor R_{GO}^{1}\cap R_{GO}^{2}\right\rfloor }{\left\lfloor R_{GO}^{1}\cup R_{GO}^{2}\right\rfloor }$$

Where ∪ and ∩ represent the intersection and union of sets, and ⌊ ⌋ represents the number of elements in the set.

(b) Sorting all the calculated distances from small to large.

(c) Calculating the percentage of positive samples in the *Y* neighbors.

In this study, the *Y* values were selected in order of 2, 4, 8, 16, 32, 64, 128, 256, and 1,024. Finally, a 10-dimensional feature vector was formed. Therefore, the digital feature vector of protein *R*_1_ can be expressed as: (*x*_1_,*x*_2,…,_ *x*_10_).

#### 3.1.2 Word Embedding

Word Embedding (16–18) is a method for converting words in text into digital vectors. The Word Embedding process was used to embed the high-dimensional space containing all the number of words into a low-dimensional continuous vector space, each word or phrase was mapped to a vector in the real number domain, and the word vector was generated as a result of the Word Embedding. In this study, we quoted the word embedding method of Qiu (33, 34). This briefly introduces how word embedding was applied in this research as described below.

Step 1: Firstly, the pupylation protein sequence was split into fragments and a wordbook is created. In this study, we used three different word embedding models, and the pupylation protein sequence is cut into different fragment lengths. Their fragment lengths can be set to 2, 3, or 4, respectively, and the step size of the moving window is 1.

Step 2: The CBOW (Continuous Bag-of-Words) model was used to train the data. In order to speed up the training speed of word vectors, the negative sampling technique (35) and backpropagation algorithm (36) were adopted in the CBOW model. At this step, the dimension sizes of the word vectors were selected as 128, 256, and 512, respectively, and we then obtained three vectors *W*_128_, *W*_256_, and *W*_512_ for a given protein sequence.

Step 3: >A protein sequence was represented by combining CBOW vectors. At this step, we merge the features of each pupylation protein sequence of the three aforementioned words vector, as shown in formula (4), and finally get an 896-dimensional vector.


(4) $$V=W_{128}⊕W_{256}⊕W_{512}$$

Among them, *W*_128_, *W*_256_, and *W*_512_ mean 128-, 256-, and 512-dimensional word vectors, and ⊕ means to concatenate a two-word vector.

### 3.2 Feature Extraction Methods for Predicting Pupylation Sites

For predicting pupylation sites, TPC (15, 21), AAI (22, 23), One-Hot (21, 37), PseAAC (25, 26), CKSAAP (21, 27, 28), and Word Embedding (17, 18) coding schemes were involved in extracting protein fragment [for example, formula (2)] information and are briefly described as follows.

#### 3.2.1 TPC

The first feature extraction algorithm applied for predicting pupylation sites in this paper is TPC (15, 21) which codes protein fragment information by calculating the frequency of occurrence of three consecutive amino acid pairs. Bian et al. (38) identified mitochondrial proteins of *Plasmodium*. In this method, we divide the number of occurrences of each of the three consecutive amino acid pairs in the fragment by the total number of all possible tripeptides [refer to formula (5)], and finally form a 9,261-dimensional digital feature vector.


(5) $$p_{i}=\frac{N_{i}}{\sum_{1}^{9261}N_{i}}$$

where *N*_i_ represents the number of occurrences of the *i*th three consecutive amino acid pairs in the fragment.

#### 3.2.2 AAI

The second algorithm, AAI code, is based on AAindex (22, 23), which is a database that collects more than 500 amino acid indexes. After evaluating the different physicochemical and biological properties of amino acids, the top 15 useful and informative amino acid indexes selected by FN Auliah et al. (15) were used in this paper (fifteen types of AAI properties can be found at https://www.mdpi.com/1422-0067/22/4/2120/s1), with a window sequence length of 57. Therefore, AAI encoding produced 855 (57 × 15) dimensional feature vectors.

#### 3.2.3 One-Hot

One-Hot coding (21, 37) is based on the 0–1 coding scheme. In this coding scheme, each amino acid is represented by a 20-dimensional binary vector. For example, alanine A is transformed into a vector (10000000000000000000), cysteine C is transformed into a vector (01000000000000000000), tyrosine Y is transformed into a vector (00000000000000000001), etc. In this study, a pseudo-amino acid code *X* was selected to represent it, which is represented by a (00000000000000000000) vector. The sequence length of the window is 57, so the total dimension of the proposed One-Hot feature vector is 20×(2δ+1), i.e., 1,140, dimensions.

#### 3.2.4 PseAAC

PseAAC (25, 26) coding has been widely used in the study of protein and protein-related problems. It can be called a “pseudo-amino acid composition” model to represent protein samples. Here, six physical and chemical properties of amino acids, hydrophobicity, hydrophilicity, molecular side chain mass, PK1, PK2, and PI, were selected to convert the protein sequence into the feature vector. The parameters ω and λ were set to 0.05 and 5, respectively [the values of ω and λ are clearly explained by Chou (39) et al.]. Finally, a 25-dimensional digital feature vector is formed.


(6) $$p_{i}=\left\{ \begin{array}{l} \frac{f_{i}}{\sum_{i=1}^{20}f_{i}+\omega \sum_{J=1}^{\lambda }\theta_{j}}\left(1\leq i\leq 20\right)\\ \frac{\omega \theta_{i-20}}{\sum_{i=1}^{20}f_{i}+\omega \sum_{j=1}^{\lambda }\theta_{j}}\left(20+1\leq i\leq 20+\lambda \right) \end{array}\right.$$

#### 3.2.5 CKSAAP

CKSAAP (21, 27, 28) coding is a coding scheme based on *K*-spaced amino acid pairs. In the coding process, a protein sequence contains 441 (21 × 21) amino acid pairs (AA, AC, AD,…, XX) and is expressed by formula (7).


(7) $$\left(\frac{F_{AA}}{F_{N}},\frac{F_{AC}}{F_{N}},\frac{F_{AD}}{F_{N}},\cdots ,\frac{F_{XX}}{F_{N}}\right)441$$

Where, *F_AA_*, *F_AC_*, *F_AD_*, *F_XX_*, represents the number of times the corresponding amino acid pair appears in the protein sequence, and *L* is used in this article to represent the length of the protein sequence, *F_N_* = *L* – *k* - 1. For each k, 441 pairs of residues are formed, where k represents the space between two amino acids, the values of *k* are 0, 1, 2, 3, 4, 5, and the best *k_max_* setting is 5. Therefore, each corresponding protein sequence can be represented with a 2,646 (21 × 21 × (*k_max_* +1)) dimensional feature vector.

### 3.3 Data Balancing and Feature Selection

In the model of pupylation protein prediction, the number of positive samples is 201 and the number of negative samples is 1126, and the ratio of positive to negative samples is approximately 1:5.6. Since it is an unbalanced data set, Random Under-sampling (RUS) (20) and Synthetic Minority Oversampling (SMOT) (19, 20) were used to process the sample data. Actually, the RUS is a very simple and popular under-sampling technique and the SMOT is one of the most popular methods in oversampling proposed by Chawla et al. (40).

In the model of pupylation site prediction, fusion of multiple features would generate a high-dimensional vector, and there may be some redundant or irrelevant features. Therefore, the chi-square test (15, 29, 30) was used to select the most beneficial feature. The chi-square test was first proposed by Karl Pearson (41), usually called the Pearson chi-square test, which is currently the most popular non-parametric(or no distribution) test based on the hypothesis of the chi-square χ^2^ distribution test method (42). In the model, the first 600-dimensional features were selected to get a better prediction result.

## 4 Model Evaluation Metrics and Operation Engine

### 4.1 Model Evaluation Metrics

In this study, four indicators were used to evaluate the performance of the model. They are Accuracy (ACC) (43), Sensitivity (SN), Specificity (SP), and Matthews Correlation Coefficient (MCC) (44–47), which are defined as Eq. (8).


(8) $$\{\begin{array}{l} Sn=\frac{TP}{TP+FN}\\ Sp=\frac{TN}{TN+FP}\\ ACC=\frac{TP+TN}{TP+FP+TN+FN}\\ MCC=\frac{TP\times TN-FP\times FN}{\sqrt{\left(TP+FP\right)\times \left(TP+FN\right)\times \left(TN+FP\right)\times \left(TN+FN\right)}} \end{array}$$

In addition, the prediction accuracy can also be measured and analyzed using the ROC curve. For the prediction method, the ROC (48) curve plots the true positive rate (Sn) and false positive rate (Sp) of all possible thresholds as a function of the relationship. The calculation of AUC also provides a comprehensive understanding of the proposed prediction method. Generally, the closer the AUC (49) value is to 1, the better the prediction method.

### 4.2 Operation Engine

Most of the classification algorithms can handle the data with the digital vector; thus, this work tried diverse approaches include Random Forest (RF), Support Vector Machine (SVM), K nearest neighbor (KNN), eXtreme Gradient Boosting (XGBoost), and Ensemble Learning. Since they have been widely used in various fields such as marketing management (50), bioinformatics (51), and image retrieval (52), we would not repeat their principles in this manuscript in detail.

In fact, the Random Forest (RF) (51, 53) algorithm is based on the classification and regression tree (CART) (54) technology which is formed by integrating multiple decision trees through the idea of integrated learning. In the RF model, each decision tree is a classifier. For a given sample, each tree will get a classification result. All the voting results are integrated, and the final output is the category with the most votes. The SVM (55) is a supervised learning model whose main idea is to find the hyperplane that distinguishes the two types, to maximize the margin, some points in the sample that are closest to the hyperplane; these points are called support vectors. The KNN (56, 57) is a supervised learning model, and its main idea is to determine which category it belongs to when predicting a new value based on the category of the nearest *K* points. XGBoost (58) is an open-source machine learning project developed by Chen et al. It efficiently implements the GBDT (59) algorithm and has made many improvements to the algorithm and engineering.

Ensemble learning (60) is an important method for improving prediction accuracy in current data mining and machine learning. It is frequently used in the field of machine learning (5) due to its “fault tolerance.” It has better classification results than individual classifiers. The ensemble method is a meta-algorithm that combines several machine learning techniques into a predictive model. There are three commonly used frameworks for ensemble learning: Bagging (61) to reduce variance, Boosting (62) to reduce bias, and Stacking (63) to improve prediction results. In this research, we used the Stacking ensemble learning algorithm. The main idea of Stacking is as follows: we firstly train multiple different models, and then use the output of each model trained before as input to train a model to get a final output. For predicting Pupylation sites, we use three base classifiers, namely, RF, SVM, and KNN, and then use LogisticRegression (LR) to classify the results of the base classification to get the final classification results.

## 5 Results and Discussion

### 5.1 Results and Discussion of Pupylation Proteins Prediction

#### 5.1.1 Effect of the Different Features

In this study, the two single feature encoding methods are GO-KNN and Word Embedding, and 10 dimensions and 896 dimensions are obtained respectively. These two kinds of features have been fused into a 906-D feature vector PUP-P-Fuse. Through the 10-fold cross-folding verification, the prediction results of different features are shown in **Table 2**.

**Table 2:** The prediction results of different feature extraction and balance methods for predicting pupylation proteins.

	Feature	ACC (%)	Sn (%)	Sp (%)	MCC	AUC
Unbalanced	GO-KNN	94.36	77.08	97.45	0.7731	0.9530
	CBOW	91.91	67.42	96.27	0.6700	0.9553
	PUP-P-Fuse	92.07	60.25	97.77	0.6615	0.9647
Balanced	PUP-P-Fuse	**95.40**	**92.03**	96.00	**0.8327**	**0.9840**

GO-KNN and CBOW represent two feature extraction methods for predicting pupylation proteins, and PUP-P-Fuse is a fusion of the above two methods.

The bold values are means the best performance of the column with the same metric and are showed in following tables with the same meaning.

From **Table 2**, we can know that the prediction results after fusion are not as good as we expected; the best prediction performance is GO-KNN’s with ACC of 94.36%, Sn of 77.08%, Sp of 97.45%, MCC of 0.7731, and AUC of 0.9530, which are slightly higher than those of CBOW and PUP-P-Fuse (see to the first 4 line of **Table 2**).

#### 5.1.2 Effect of the RUS and SMOTE

Using Random Under-sampling (RUS) and Synthetic Minority Over-sampling (SMOTE) to balance the data, and then through 10-fold cross-folding verification, the prediction results of ACC, Sn, Sp, MCC, and AUC on balanced and unbalanced data sets were obtained and are shown in **Table 2**.

**Figure 2:**
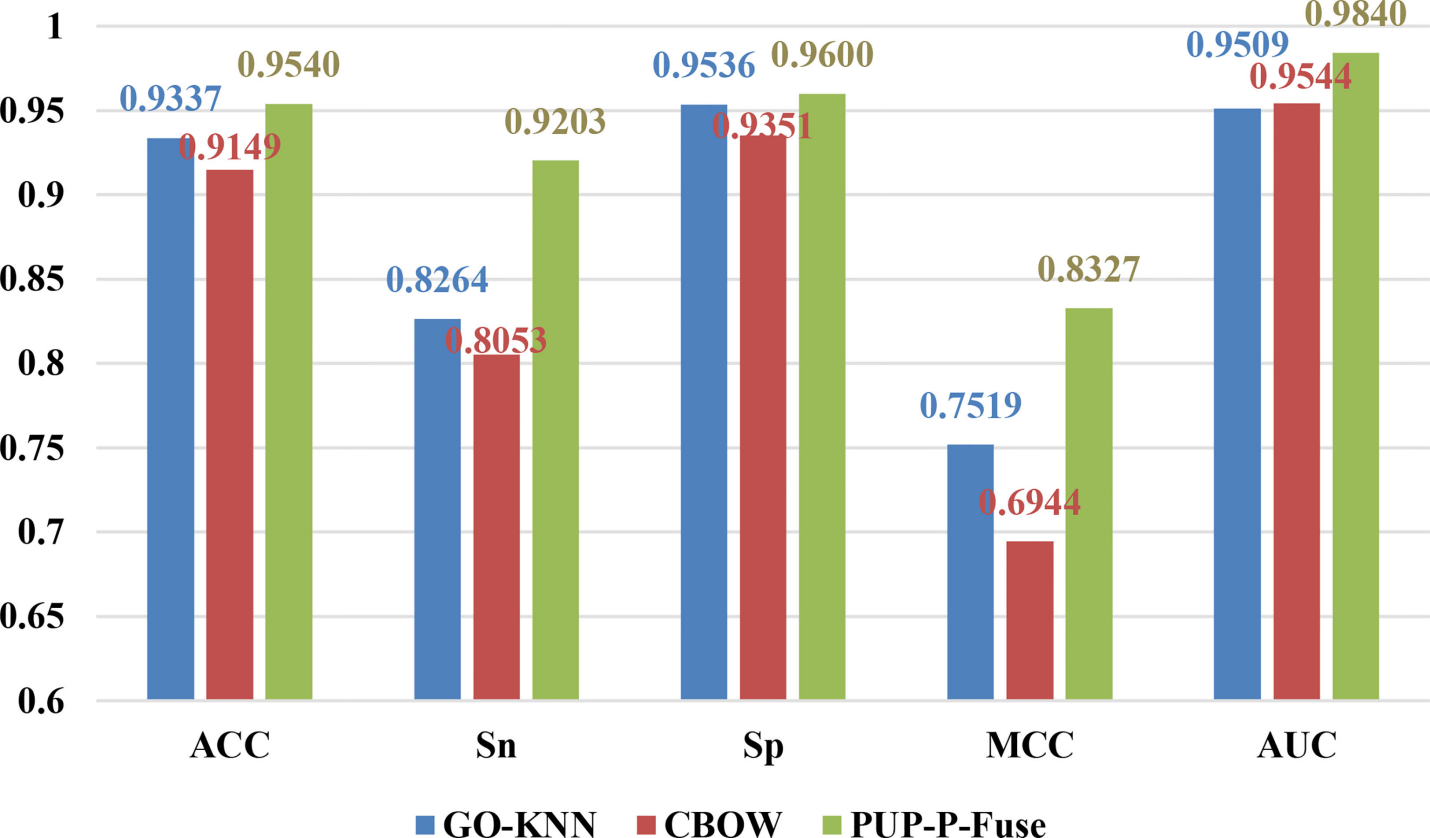
The prediction results of different characteristics on balanced data for predicting pupylation proteins.

From the last line of **Table 2**, we can see that the PUP-P-Fuse’s ACC, Sn, MCC, and AUC predictive indicators have increased by 3%, 32%, 17%, and 2%, respectively, after the RUS and SMOTE technology balance. Therefore, the results show that multifeature fusion (PUP-P-Fuse) can improve the performance. In order to better analyze the influence of different features on pupylation protein prediction, the results obtained by two single coding and fusion features are as shown in **Figure 2**.

From **Figure 2**, we can see that the ACC, Sn, Sp, MCC, and AUC of GO-KNN are 93.37%, 82.64%, 95.36%, 0.7519, and 0.9509, respectively. Those of CBOW and PUP-P-Fuse are denoted with red and green bars, respectively. Compared with GO-KNN and CBOW’s ACC, Sn, Sp, MCC, and AUC predictive indicators, the PUP-P-Fuse increased by 2%–4%, 10%–11%, 0.6%–2%, 8%–13%, and 3%, respectively. In summary, all indicators of PUP-P-Fuse are higher than the other two models after data balancing. Therefore, it is proper to use RUS and SMOT in this issue.

#### 5.1.3 Effect of Classifiers

**Figure 3:**
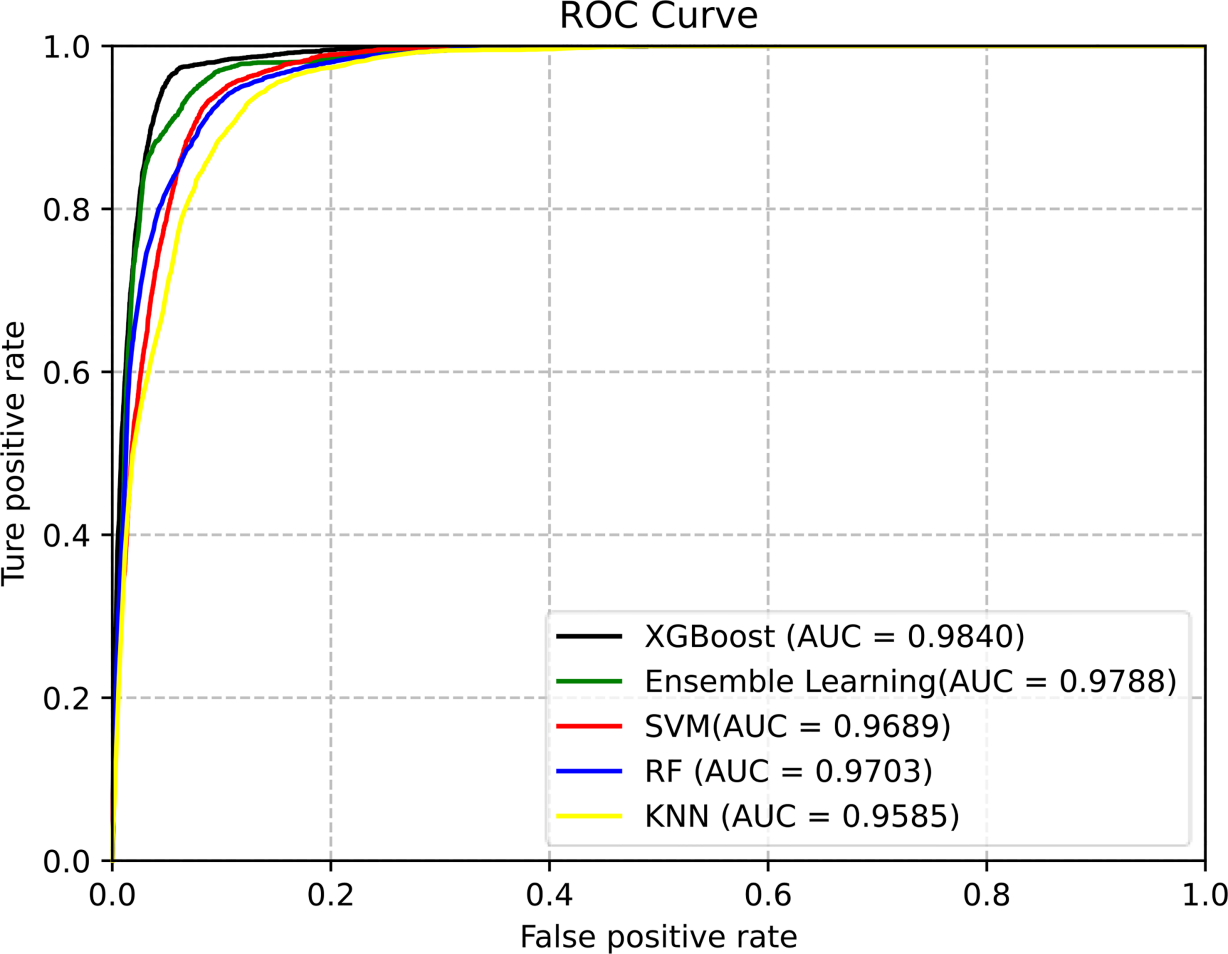
ROC curves of different classifiers for predicting the pupylation protein.

Classifiers play an important role in prediction. In this work, we used the above five classifiers to identify pupylation proteins. After 10-fold cross-folding verification, the results of ACC, Sn, Sp, MCC, and AUC of each classifier are shown in **Table 3**. From **Table 3**, we can see that XGBoost gained the best performance on each evaluation index. In order to better compare the effects of different classifiers, the prediction results of the five classifiers are as shown in **Figure 3**.

**Table 3:** The prediction results of different classifiers for predicting pupylation proteins.

Algorithms	Acc (%)	Sn (%)	Sp (%)	MCC	AUC
XGBoost	**95.40**	**92.03**	**96.00**	**0.8327**	**0.9840**
Ensemble Learning	93.87	90.61	94.48	0.7874	0.9788
SVM	91.36	93.65	90.96	0.7335	0.9689
RF	92.87	82.40	94.75	0.7355	0.9703
KNN	83.88	96.90	81.55	0.6104	0.9585

The bold values are means the best performance of the column with the same metric and are showed in following tables with the same meaning.

The area under the ROC curve can evaluate the predictive performance of the model. It is seen in **Figure 3** that the XGBoost classifier, of which AUC is 0.9840, is the best choice for the proposed model.

#### 5.1.4 Effect of Features on the Independent Dataset

To verify the effect of the PUP-P-Fuse model, we used 67 pupylation proteins and 134 negative samples for independent testing; PUP-P-Fuse has the highest performance, as shown in **Table 4**. It can be seen that the effect of the PUP-P-Fuse model is still very good. However, from **Table 4** we can see that the overall performance of the SVM classifier is better than those of other classifiers.

**Table 4:** The prediction results of different classifiers on the testing set of pupylation proteins.

Algorithms	Acc (%)	Sn (%)	Sp (%)	MCC	AUC
XGBoost	84.66	80.99	86.62	0.6630	0.9251
Ensemble Learning	85.34	80.96	87.41	0.6738	0.9376
SVM	85.48	88.78	83.85	0.6955	0.9317
RF	84.55	79.15	87.79	0.6571	0.9270
KNN	78.56	83.97	75.61	0.5653	0.8868

### 5.2 Results and Discussion of Pupylation Site Prediction

#### 5.2.1 Effect of Features on the Training Dataset

In this study, six single-feature codes are AAI, One-Hot, PseAAC, Word Embedding, CKSAAP, and TPC, and the feature PUP-S-Fuse was obtained after fusion. The six features are coded separately and obtained 855, 1140, 26, 896, and 2,646 dimensions, respectively. Through 10-fold cross-folding verification, we choose the SVM classifier for training. Without feature selection, we obtain the prediction results of different feature extractions with a ratio of positive samples to negative samples of 1:1, as shown in **Table 5**.

**Table 5:** The effect of different feature extraction methods on the training set of pupylation sites.

Features	ACC (%)	Sn (%)	Sp (%)	MCC	AUC
AAI	56.71	56.21	57.52	0.1380	0.6148
One-Hot	57.49	59.49	55.95	0.1550	0.6296
PseAAC	61.56	62.00	61.64	0.2367	0.6597
Word Embedding	69.92	73.36	66.55	0.4001	0.7645
CKSAAP	68.84	68.92	69.20	0.3818	0.7596
TPC	70.36	70.69	70.65	0.4143	0.7697
PUP-S-Fuse	**74.00**	**80.00**	**68.55**	**0.4883**	**0.7951**

The bold values are means the best performance of the column with the same metric and are showed in following tables with the same meaning.

From **Table 5**, we can see that the ACC, Sp, MCC, and AUC indicators of TPC are all higher than other single codes, and the Sn indicators of Word Embedding are all higher than other single codes. The fusion feature code PUP-S-Fuse performs better than any single feature on ACC, Sn, Sp, MCC, and AUC indicators. Therefore, feature fusion is very necessary for this issue.

#### 5.2.2 Effect of the Chi-Square Test on the Training Dataset

As regards the model for predicting the pupylation site, we selected different *K* values for the chi-square test and compared them and found that the prediction effect has been relatively greatly improved after the chi-square test was used to select features.

It is seen in **Table 6** that when the *K* value is selected as 600, the ACC, Sn, and MCC of the pupylation site are predicted to be higher than other *K* values. When the *K* value is selected as 1,000, the Sp and AUC values of the pupylation site are higher than those of other *K* values. Therefore, from the overall effect, we finally selected 600 for predicting the pupylation site.

**Table 6:** The effect of feature fusion Pup-S-Fuse by using the chi-square test for predicting pupylation sites.

Features	ACC (%)	Sn (%)	Sp (%)	MCC	AUC
K = 200	89.09	88.82	89.57	0.7830	0.9531
K = 400	91.21	92.89	89.52	0.8256	0.9565
**K = 600**	**92.30**	**93.97**	**90.71**	**0.8477**	0.9599
K = 800	91.99	93.31	90.55	0.8400	0.9634
K = 1,000	92.00	92.27	**91.78**	0.8394	**0.9641**
K = 1,200	90.70	91.77	89.70	0.8145	0.9604

The bold values are means the best performance of the column with the same metric and are showed in following tables with the same meaning.

#### 5.2.3 Effect of Classifiers on the Training Dataset

Choosing the right machine learning (ML) algorithm is also a crucial step for predicting results. When predicting pupylation sites, we used RF, SVM, KNN, Ensemble Learning (EL), and XGBoost algorithms. In order to verify the effectiveness and superiority of the EL algorithm used to predict pupylation sites, we compared these algorithms through 10-fold cross-validation on the same training set. The prediction results are shown in **Table 7**.

**Table 7:** The prediction results of different classifiers for predicting pupylation sites.

Algorithms	Acc (%)	Sn (%)	Sp (%)	MCC	AUC
EL	**92.30**	93.97	**9071**	**0.8477**	0.9599
SVM	91.72	**95.27**	88.59	0.8377	**0.9659**
RF	86.72	87.50	86.24	0.7361	0.9347
KNN	81.34	90.37	75.63	0.6706	0.9388
XGBoost	78.49	79.02	77.94	0.5703	0.8622

EL, ensemble learning.

The bold values are means the best performance of the column with the same metric and are showed in following tables with the same meaning.

**Figure 4:**
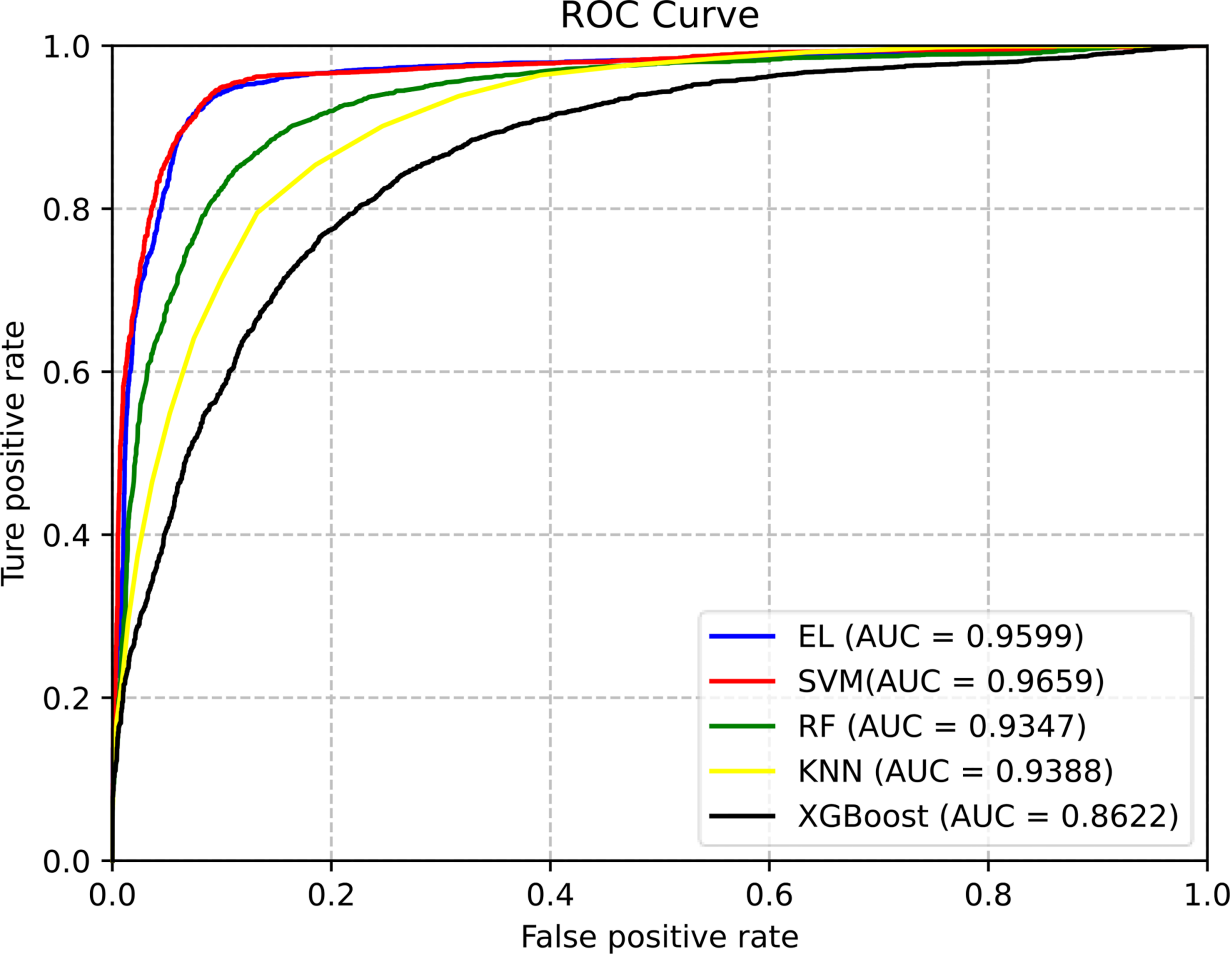
The ROC curves of different classification methods for predicting pupylation sites. (EL is the abbreviation of ensemble learning).

From **Table 7**, although we know that the prediction effect of the EL classifier and SVM classifier is better, the overall prediction effect of the EL is better than that of the SVM. The prediction results of RF, KNN, and XGBoost are relatively poor. In order to evaluate the performance of the classifier more comprehensively, the ROC curves of different classifiers are as shown in **Figure 4**.

From **Figure 4**, we can clearly see that the area under the ROC curve of EL and SVM is the largest, and the AUC of EL is about 2%–10% higher than that of other ML models. Therefore, EL was selected as the best classifier for predicting pupylation sites.

#### 5.2.4 Comparison With Other Methods on Independent Datasets

In order to compare PUP-S-Fuse with the existing five methods (GPS-PUP, iPUP, PUPS, PbPUP, and PUP-Fuse), tests were performed on the same independent set which contains 86 pupylation sites and 1,136 non-pupylation sites from 71 pupylation proteins. PUP-S-Fuse and PUP-Fuse were trained with the same training data set mentioned above, and the other four methods were quoted from the references. In the fairly compared performance, PUP-S-Fuse provided the highest performance, as shown in **Table 8**.

**Table 8:** Comparison of methods on Independent Dataset for predicting pupylation sites.

Methods	Acc (%)	Sn (%)	Sp (%)	MCC	AUC
iPUP	73	40	88	0.32	
GPS-PUP	68	21	89	0.13	
PUPS	67	17	89	0.08	
pbPUP	79	48	82	0.45	
PUP-Fuse	82	59	91	0.55	
PUP-S-Fuse	**91.35**	**78.26**	**97.38**	**0.7953**	**0.9550**

The bold values are means the best performance of the column with the same metric and are showed in following tables with the same meaning.

From **Table 8**, we know that the performance of PUP-S-Fuse on the test set is also better than that of PUP-Fuse. Acc, Sn, Sp, and MCC are increased by 9%, 19%, 6%, and 24%, respectively, which proves that PUP-S-Fuse is superior to existing predictors.

## 6 Web Server and User Guide

The actual application value of a prediction method can be significantly improved if it has a web server that can be viewed by the public; accordingly, the PUP-PS-Fuse web server has been established. To maximize the convenience of most experimental scientists, a guide for users is provided below.

Step 1. Opening the web server at “https://bioinfo.jcu.edu.cn/PUP-PS-Fuse,” the server consists of four main modules, namely, Pupylation Protein, Pupylation Site, Download (data download), and **Help** (website usage guide). You will see the top page of PUP-PS-Fuse on your computer screen.

Step 2. In the Pupylation Protein prediction module, you can enter the protein sequence in the input file box, but it must be in FASTA format. You can also click the example button where you will see that there are a correct example and an incorrect example as well as the text input format. Click the Close button, and you will return to the pupylation Protein prediction interface. Click the Submit button to get the prediction results. After 20 seconds or so since your submitting, you will see the following on the screen of your computer: “The Pupylation protein list includes …” and “The non-Pupylation protein list includes …”

Step 3. In the Pupylation Site prediction module, you can enter the protein sequence in FASTA format in the input file box. In the example_site submodule, you will see that there are a correct example and an incorrect example as well as the text input format. Click the Close button, and you will return to the pupylation Site prediction interface. Click the Submit button to get the predicted results. After 2 min or so since your submitting, you will see the following on the screen of your computer: ‘The number of “K” is X. Location M_1_, M_2_, M_3_, … is(are) predicted to be Pupylation Site(s).’

In the Download module, you can download the Pupylation protein dataset and Pupylation site dataset (also available in the **Supplementary Material**). By the way, you can click on the Help button to see a brief introduction about the predictors.

## 7 Conclusion

PUP-PS-Fuse was developed to predict pupylation proteins and sites. In order to predict pupylation proteins, GO-KNN and Word Embedding served as feature extraction methods. In the work, GO-KNN extracted features based on the KNN score matrix of functional domain GO annotations, and Word Embedding converted information of the amino acid sequence into digital feature vectors. In addition, RUS and SMOT technology were used to deal with the imbalance of the data set to reduce the negative impact of imbalance on the model. Finally, the XGBoost classifier was selected to make predictions. In order to predict pupylation sites, six feature extraction codes and one fusion feature extraction code are used, named as TPC, AAI, One-Hot, PseAAC, CKSAAP, Word Embedding, and PUP-S-Fuse. In order to improve the computational efficiency and eliminate the redundancy and noise generated by the fusion feature, the chi-square test served to reduce the dimensionality of the fusion feature. The selected feature subset was input into the Ensemble Learning for classification, and then 10-fold cross-folding was used for verification. The performance of PUP-S-Fuse is evaluated based on an independent test data set, and compared with other existing methods, it is concluded that the predictive performance of PUP-S-Fuse is better than other existing methods. These processes only require calculation models and do not require any physical and chemical experiments, which saves experimental costs and improves work efficiency. We hope that this work will be helpful for dealing with some related biological problems with computational methods.

## Data Availability Statement

The original contributions presented in the study are included in the article/**Supplementary Material**. Further inquiries can be directed to the corresponding authors.

## Author Contributions

W-RQ conceived and designed the experiments. M-YG, Q-KW, and L-LL performed the extraction of features, model construction, model training, and evaluation. M-YG drafted the manuscript. XX and W-RQ supervised this project and revised the manuscript. All authors contributed to the article and approved the submitted version.

## Funding

This work was supported by grants from the National Natural Science Foundation of China (Nos. 31760315, 62162032, 61761023) and Natural Science Foundation of Jiangxi Province, China (No. 20202BAB202007).

## Conflict of Interest

The authors declare that the research was conducted in the absence of any commercial or financial relationships that could be construed as a potential conflict of interest.

## Publisher’s Note

All claims expressed in this article are solely those of the authors and do not necessarily represent those of their affiliated organizations, or those of the publisher, the editors and the reviewers. Any product that may be evaluated in this article, or claim that may be made by its manufacturer, is not guaranteed or endorsed by the publisher.
